# Risk Perception & Risk-Reduction Behavior Model for Blue-Collar Workers: Adapted From the Health Promotion Model

**DOI:** 10.3389/fpsyg.2020.538198

**Published:** 2020-11-04

**Authors:** Won Ju Hwang, Mi Jeong Kim

**Affiliations:** ^1^College of Nursing Science, East-West Nursing Research Institute, Kyung Hee University, Seoul, South Korea; ^2^School of Architecture, Hanyang University, Seoul, South Korea

**Keywords:** workers, theory evaluation, health promotion model, cardiovascular disease, risk perception

## Abstract

This study examined the health promotion model (HPM) as a framework for assessing perceptions and health-related behaviors related to cardiovascular disease (CVD) risk among blue-collar workers. This was done with the aim of providing time-sensitive educational and training materials for workers while on the job or functioning in their communities. The revised HPM was evaluated in the above context using specific criteria developed by Chinn and Kramer (2008) and scoping literature review. Specifically, we assessed the model based on five criteria such as its clarity, simplicity, generality, accessibility, and importance. The revised HPM showed strengths in both accessibility and generality. That is, it applied to all populations and chronic illnesses through clearly defined and specified major concepts. However, there were several weaknesses in areas of clarity and consistency; the model included three new concepts (i.e., activity-related affect, commitment to a plan of action, and immediate competing demands and preference) that actually decreased these elements. In this context, situational influences require adequately reflected external variables. Nevertheless, the revised HPM showed predictive power among this study’s target population. The HPM was modified to address deficiencies in regard to the concept of risk perception. Work-related situational influences were also restructured based on individual and environmental characteristics. The modified framework can be used to clarify health-related behaviors among blue-collar workers.

## Introduction

While all workers are affected, those with blue-collar jobs are particularly at high risk for cardiovascular disease (CVD) due to overtime, occupational physical activity, and job stress (Hwang et al., 2015). When compared to white-collar workers, the incidence rate of CVD among blue-collar workers has also increased in recent decades (Hwang et al., 2015). It is thus imperative to provide appropriate treatments for these workers by investigating their perceived CVD risks, risk-reduction behaviors, and knowledge of CVD. However, little is currently known about either risk perception or risk-reduction behaviors among blue-collar employees (Doran et al., 2018; Hwang and Kim, 2019). There is also a lack of evidence related to the environmental, psychosocial, organizational, and individual factors that influence risk perceptions and risk-reduction behaviors in this context.

Previous research has shown that perceived CVD risk is positively associated with the desire to make risk-reduction behavioral changes (Amarasekara et al., 2016; Fang et al., 2019; Haynes et al., 2019). However, meta-analytic studies on risk perception have pointed out that the associations between risk perception and health behaviors are not particularly strong (Harrison et al., 1992; Brewer et al., 2007; Ayaz-Alkaya et al., 2020). It is likely that health behavior is a complex phenomenon that is influenced by multiple factors, including risk perception. As such, the associations between risk perception and CVD risk factors require continued theoretical examination in order to gather information for use in time-sensitive treatments and interventions designed for workers while on the job or in the community.

Pender’s (1984) health promotion model (HPM) is a nursing-based framework designed to predict health behaviors. Following social learning theory, a revised HPM was created to identify the factors associated with exercise behaviors, which promote health through personal and behaviorally specific cognitions and affect. Behavior-specific cognitions and affect are categories of major motivational significance that are critical for interventions due to their modifiability (World Health Organization, 2008; Heydari and Khorashadizadeh, 2014).

This study examined the theories used to understand CVD risk perceptions and health-related behaviors among workers, with a specific focus on blue-collar jobs. In this context, the HPM is frequently used to capture the health behaviors of individual workers. As such, this study specifically aimed to (a) review and critically evaluate the HPM following criteria established by Chinn and Kramer (2008), (b) illustrate the theoretical elements of the HPM through a review of previous studies, and (c) create a modified framework based on the HPM. The next section discusses the specific criteria used for the critical analysis.

## Analysis Method

The HPM was evaluated using criteria developed by Chinn and Kramer (2008) as well as based on the scoping review (Munn et al., 2018). The critical reflection approach facilitates a better understanding of how well a theory applies in practice, research, and/or educational activities.

Considering that a given theory is primarily designed to produce a deeper understanding of phenomena and guide related research, this study used the following questions in its critical analysis:

How (a) clear, (b) simple, (c) general, (d) accessible, and (e) important is the theory?

As such, these were the criteria used to evaluate the HPM. The following section introduces a brief description of the theory behind the HPM in addition to its definition, associated concepts, assumptions, and propositions. This is followed by a discussion of the abovementioned five criteria (clarity and consistency, simplicity, generality, accessibility, and importance).

Additionally, the scoping literature review identified papers in terms of the accessibility of HPM.

**FIGURE 1:**
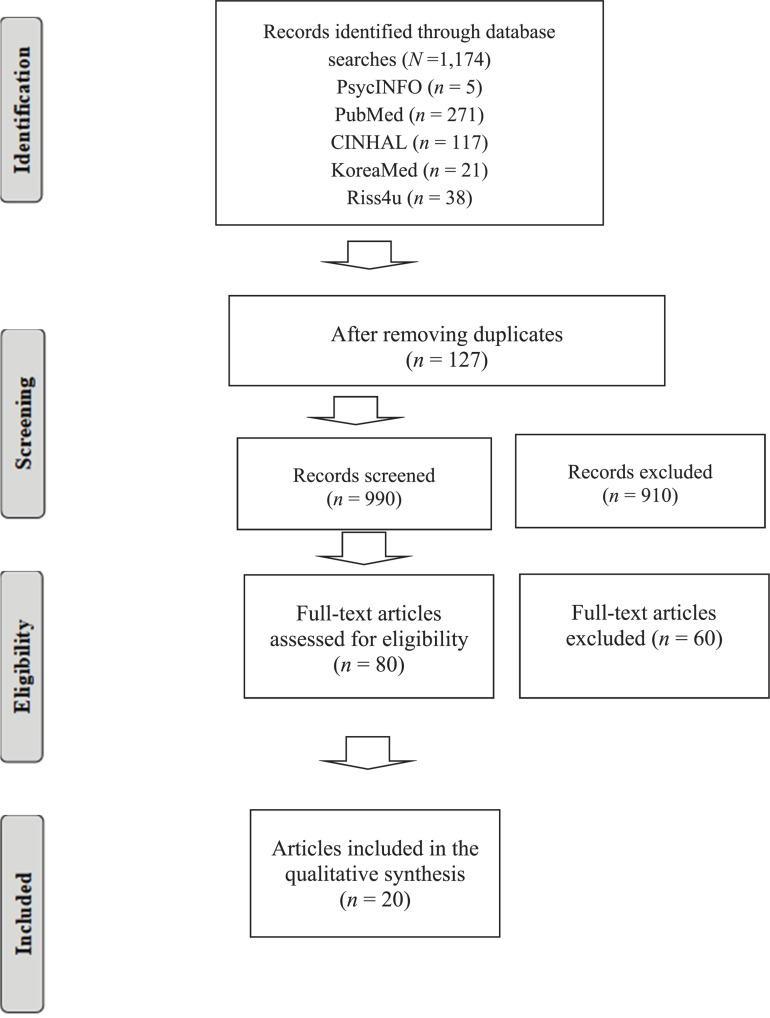
Flow diagram of study selection for the scoping review.

Scoping review frameworks assist in mapping out particular research areas of interest and allow reviewers to examine the extent and nature of the evidence available. Scoping literature reviews differ from systematic reviews in that the process is not fixed, enabling reviewers to redefine selection criteria based on the findings of the initial search strategy (Munn et al., 2018; Figure 1).

The search was limited to studies published in English and Korean. The following keywords and Medical Subject Headings were used: “Theory,” “Health promotion model,” “concept,” “health-related behavior,” “risk reduction,” “health behavior,” “health promotion,” “disease prevention,” “health behavior,” “health promotion,” “worker,” and “occupation.” The study outcomes selected were health-related behavior.

As the data used in this scopic review were obtained from previously published studies, ethical approval and consent were not required. The inclusion criteria were as follows: studies about HPM; studies that applied health promotion concept; and studies published in English or Korean. Intervention studies that solely focused on pharmacological therapy was excluded.

## Results

### Description of HPM

Health promotion model was designed to depict the multidimensional nature of individuals as they engage in surrounding interpersonal and physical environments while pursuing health promotion. The model specifically focuses on activities that are aimed at increasing well-being and optimizing health status for these individuals as well as their families, communities (e.g., worksites), and general society. The HPM thus provides a framework for understanding the various forces that influence individuals while seeking to improve their health.

**FIGURE 2:**
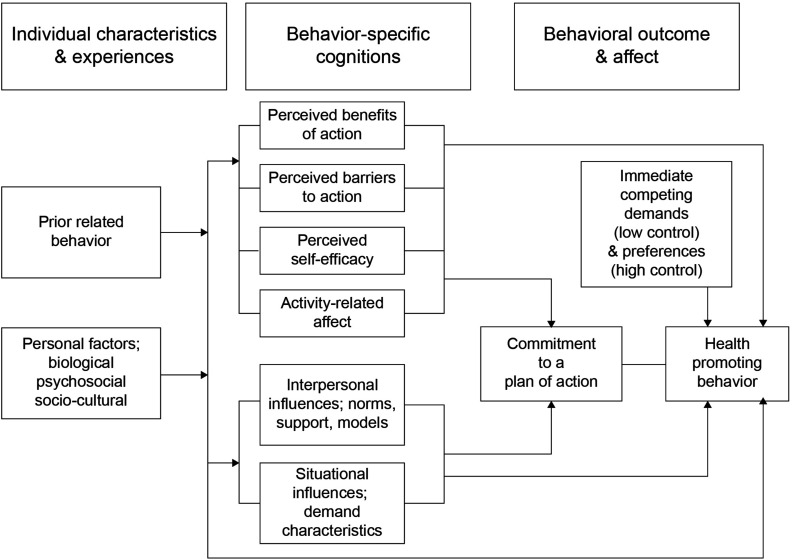
Pender’s health promotion model. (Previous model). Adopted from Pender et al. (2006).

Figure 2 presents a flowchart for the HPM. As shown, the model consists of the three following constructs: (a) individual characteristics and experiences, (b) behavior-specific cognitions and affect, and (c) behavioral outcome. The model entails that individual characteristics and experiences influence behavior specific cognitions and affect, thus leading to a committed plan of action. Such commitment then results in the behavioral outcome of practicing health-promoting behavior. However, Pender (1984) posited that competing demands and preferences may influence and modify the outcome. That is, all predictive variables directly influence the behavioral outcome.

#### Assumptions and Propositions

The primary assumption of the HPM is grounded in the idea that individuals are responsible for shaping and maintaining their own health behaviors. On the other hand, the HPM’s concepts are rooted in its theoretical propositions. The key exemplar propositions are as follows: (a) perceived barriers may constrain one’s commitment to action and actual behavior, (b) perceived competence and self-efficacy when executing a given behavior increase the likelihood of behavioral performance, and (c) situational influences in the external environment may influence one’s level of commitment to participate in health-promoting behavior (Oh and Yi, 2017). The theory further entails that individuals are likely to commit to behaviors they believe will result in personally valued benefits.

### Critical Reflections on the Health Promotion Model

#### Clarity and Consistency

##### Semantic clarity

The HPM achieves semantic clarity by providing both specific and general traits for its major concepts. Psychosocial factors include variables such as self-esteem, self-motivation, and perceived health status (Pender et al., 2006). However, the concepts of “commitment to a plan of action” and “immediate competing demands and preferences” require further clarification. Specifically, the theory does not provide an adequate theoretical definition for commitment to a plan of action, instead stating that its value is difficult to quantify.

Pender stated that situational influences included one’s perceptions of the available options, demand characteristics, and the environment in which a given health-promoting behavior would occur (Joseph-Shehu et al., 2019). While the theory behind this provides specified components for situational influences, there are no detailed descriptions of the demand characteristics or environment. Further, use of the term “demand characteristics” may introduce uncertainty because such characteristics may include both internal and external components. In addition, “the environment” can include fascinating and/or uninteresting components as well as objective and subjective characteristics. As such, appropriate theoretical definitions would facilitate a better differentiation among the components involved in situational influences.

##### Structural clarity

The HPM emphasizes the detailed relationships among the three constructs (including the possible influences of behavior-specific cognitions and affect on behavioral outcomes) through the diagram shown in Figure 1. In this context, the theory propositions are clearly stated. However, the possible relationship between perceived benefits and barriers is missing. For example, perceived benefits may be stronger predictors of behavior change when perceived barriers are low. Alternatively, individuals may not perform appropriate actions even if the perceived benefits are high under a condition involving high perceived barriers. Thus, perceived benefits and barriers influence each other. Further, commitment to a plan of action as detailed in the revised HPM also implies an underlying cognitive process; although it may be more closely related to factors of cognition, it is actually structured for behavioral outcomes.

##### Semantic consistency

There is an issue of consistency in regard to several factors involved in understanding the HPM. First, three new variables were added to the revised model (i.e., activity-related affect, commitment to a plan of action, and immediate competing demands and preferences). These concepts are somewhat problematic in semantic clarity and may affect semantic consistency because their theoretical definitions are unclear. Second, a significant inconsistency was found between the conceptual behavioral factors and prior related behavior. That is, behavioral factors were retained in the HPM as “prior related behavior.” Pender (1984) proposed that prior behavior entailed both direct and indirect effects on the likelihood of engaging in health-promoting behavior and was the best predictor. However, the HPM concludes that behavior-specific cognitions are the main predictive factors for health outcomes while ignoring the effects of prior related behavior as suggested earlier. Further, behavioral factors solely posed indirect effects on health-promoting behavior prior to the change in conceptual labeling (i.e., behavioral factors changed to prior related behavior).

##### Structural consistency

Another problem of inconsistency can be found when looking at the concepts of interpersonal and situational influences. Specifically, the original model showed these two concepts in relation to modifying factors; however, they were relocated to behavior-specific cognition and affect factors in the revised model. Interpersonal influences and situational influences were not considered cognition factors in the original model. However, these concepts also indirectly influence health-promoting behavior. Pender (1984) stated that interpersonal and situational influences were reconceptualized in the revised model as direct influences on health behavior. Specifically, situational influences (which are considered external influences) may both directly and indirectly affect an individual’s behaviors through perceived self-efficacy by presenting a given environment. Therefore, the influences of interpersonal and situational factors on health-related behavior are not used in the original context. A critical lack of structural consistency is noted in this regard.

#### Simplicity

The HPM incorporates multiple factors to explain health-promoting behavior. However, it uses relatively few (i.e., 11) to address the complex phenomenon of health-promoting behavior. Indeed, it seems to have achieved a balance between thoroughness and parsimony. However, the relationships within behavior-specific cognitions and affect are extremely complex because many concepts and their internal or external relationships are included in the model. The model retains some level of complexity because it includes relationships among multiple components and also within each component. There is further potential complexity because a number of sub-concepts are contained within each concept. The implicit effects of each concept and their various links to behavioral outcomes thus contribute to the model’s overall complexity.

The pathways presented between the six concepts and behavior-specific cognitions and affect seem to simplify their relationships (Figure 2). Based on the diagram, the HPM assumes directional relationships between activity-related affect, self-efficacy, and perceived barriers. However, the relationships underlying those cognition factors may be more complex. For example, self-efficacy always precedes perceived barriers, while perceived benefits may also be affected by other cognition factors (e.g., perceived barriers and self-efficacy). Further, the HPM does not consider cognitive factors other than activity-related affect, self-efficacy, and perceived barriers. Although the large groupings illustrate the level of interrelation between factors, there are no specific explanations of these relationships. To provide a more complete understanding of the active cognitive factors, the HPM should thus illustrate the pathways among these and explain their relationships to health-promoting behaviors. In addition, the relationship between perceived benefits and barriers to action is missing. However, the model proposes that perceived barriers influence perceived benefits to action, with higher barriers resulting in lower perceptions of the benefits to completing the considered health behavior.

#### Generality

The HPM is relatively broad when compared to other behavioral models as it includes a number of intrapersonal factors (perceived barriers to the behavior), interpersonal factors (social support), and situational influences (availability of healthful options). It also has implications for health promotion in the workplace and community. Further, the HPM can be applied to almost any population (e.g., women, youths, the elderly, and workers) throughout multiple developmental stages (McCullagh et al., 2002; Wu and Pender, 2002; Naumanen, 2006; Coulombe et al., 2017; Joseph-Shehu et al., 2019) and among patients with chronic diseases (Carson et al., 2014; Oh and Yi, 2017; Yen and Li, 2019). It is also useful for understanding the psychosocial and environmental effects on health-promoting behavior in both the academic and practical settings due to a complex look at the relationships between health-promoting behavior and the importance of various related factors. Moreover, it is valuable for studying risk reduction in a variety of community settings, especially the workplace.

#### Accessibility

The following literature review discusses 20 studies that tested the major propositions of the HPM between 1990 and 2019. Among these studies, five included industrial workers (Pender et al., 1990; Lusk et al., 1999; McCullagh et al., 2002; Hwang et al., 2015). However, only one included CVD risk and risk-reduction behavior as a primary outcome (Williams et al., 2004; see Table 1).

**TABLE 1:** Summary of research studies using the health promotion model.

Investigator (year)	Sample (n)	Framework, design, and purpose	Measurement	Major findings
Pender et al. (1990)	Workers enrolled in health-promotion programs (589)	– Quasi-experimental study – To evaluate a health promoting lifestyle	– HPLP II –Outcome: health promoting behavior (risk reduction behavior)	Perceived personal competence, definition of health, perceived health status, and perceived control of health accounted for 31% of the variance in health-promoting lifestyle patterns.
Lusk et al. (1999)	Construction workers (356)	– Descriptive study – To investigate the strongest predictors from the HPM	– Outcome variable: use of hearing protection devices	Five predictors had statistically significant regression coefficients: perceived noise exposure, self-efficacy, value of use, barriers to use, and modeling of use of hearing protection.
Coulombe et al. (2017)	Workers of labor unions in Canada (669)	-A quantitative, cross-sectional research – To promote men’s health.	-Health-Promoting Neighborhood Questionnaire (HPNQ)	The HPNQ can be a useful tool to monitor men’s perceptions of their home and workplace neighborhoods
Joseph-Shehu et al. (2019)	University staff (22)	– Quasi-experimental study – To develop and pilot test an Integrated Technology–Moderated Health Promotion Model	– HPLP-II – Outcome: health-promoting lifestyle behaviors (HPLB)	The result of the pilot testing of the model showed that the model enhances health-promoting lifestyle behaviors and improves the health status of staff.
Seo and Ha (2019)	College students (264)	– A quantitative, cross-sectional research – To identify factors influencing PA in male and female college students based on the Health Promotion Model.	– HPLP II (52 items)	A multiple regression analysis indicated that the factors affecting PA in male college students were PA self-efficacy and subjective economic status, while the factors affecting PA in female students were PA self-efficacy, subjective health status, activity-related affect, and peer support.
Voskuil et al. (2019)	Girls in the 5th–8th grades (517)	– Randomized controlled trial (RCT) – To test hypothesized relationships of the health promotion model (HPM) as a means of predicting moderate-to-vigorous physical activity (MVPA) among urban, adolescent girls.	behavior-specific cognitions and effect, and the behavioral outcome of MVPA	The sample represented a diverse group of girls with 233 (45.1%) indicating black race, 146 (28.2%) indicating white race, 57 (11.0%) indicating mixed race with 48 (84.2%) of those girls selecting black as part of a mixed race, and 87 (16.8%) indicating Hispanic ethnicity.
Martinelli (1999)	Young adults, aged 18–25 (136)	– Cross-sectional study –To test an explanatory model on the avoidance of environmental tobacco smoke (ETS)	– General self-efficacy scale, HPLP, and ETS avoidance scale	26% of the variance in avoiding ETS was accounted for by gender, having self-efficacy, and ETS-avoidance efficacy, not living with people who smoke, and performing other healthy behaviors.
Oliver-Mcneil and Artinian (2002)	Women with newly diagnosed CHD, mean age 65.6 (33)	– Descriptive study – To describe CVD risk perceptions and behavior	– HPLP II – Outcome: risk-reduction behavior	The risks identified were considerably fewer and differed from those documented in the women’s medical records.
McCullagh et al. (2002)	Farmers (139)	– Descriptive study – To understand factors influencing the use of hearing protection devices	– Modified instruments – Outcome: hearing protection device use	Interpersonal support, barriers, and situational influences as statistically significant predictors of this health behavior, correctly predicting 78% of the cases.
Penwell-Waines et al. (2017)	adults with confirmed MS diagnoses who were receiving care at Augusta MS Center (121)	– A quantitative, cross-sectional research – To apply the HPM to better understand adherence and QoL in PwMS	Health-promoting behaviors	Adherence Variables included in the HPM accounted for a small portion of variance for the adherence outcome. Quality of life was more fully explained by the HPM model than was adherence (*F* = 26.92, *p* < 0.001). In the full model, anxiety, depression, stigma, and self-efficacy were the strongest variables.
Kamran et al. (2015)	Hypertensive adults (671)	– Cross-sectional study – To examine the relationships between the health promotion model (HPM) constructs and sodium intake, and to determine the predictive power of the HPM constructs in rural Iranian hypertensive patients.	– HPM	Sodium intake was negatively correlated with perceived benefits, perceived self-efficacy, situational influences, interpersonal influences, commitment to action, affects related behavior, and positively associated with the perceived barriers score. The structural equation modeling showed that the model explained 63.0% of the variation in sodium intake
Eren Fidanci et al. (2017)	86 obese children and their parents (48 in the experimental group and 38 in the control group)	– Quasi-experimental study – To determine the effects of an intervention based on Pender’s Health Promotion Model (HPM) on the healthy life behaviors and self-confidence of obese children.	– HPM – Healthy life behaviors	Experimental group participants showed a significant increase in healthy eating habits such as noting food portions and choosing water instead of sugary drinks and spent significantly less time in front of a television or computer. Furthermore, experimental group participants had reduced their total body mass index standard deviation score and had an average self-confidence score that differed from the control group.
Sohng et al. (2002)	Elderly Korean immigrants, mean age 75.5 (range 60–89) (110)	– Cross-sectional study – To examine the relationship between health promoting behaviors, perceived health status, and self-efficacy	– HPLP (Korean version, 42 items)	Self-efficacy (*r* = 0.49, *p* = 0.01) and perceived health status (*r* = 0.19, *p* = 0.043) were significantly related to health-promoting behaviors.
Tang and Chen (2002)	Caregivers, mean age 52.2 ± 14.6 (range 21 – 90) (541)	– Descriptive study – To examine health promotion practice	– HPLP II (52 items)	Indicated that greater participation in health promoting practice was associated with higher education (*r* = 0.26, *p* < 0.01), higher income (*r* = 0.26, *p* < 0.05), and higher perceived health status (*r* = 0.40, *p* < 0.01).
Wu and Pender (2002)	Adults (832)	– Cross-sectional study – To determine health promotion practice	– Cognitions (self-efficacy, perceived benefits/barriers)	Indicated that perceived self-efficacy (*r* = 0.46, *p* < 0.01), perceived benefits (*r* = 0.31, *p* < 0.01), and perceived barriers (*r* = 0.46, *p* < 0.01) were significantly related to physical activity.
Hulme et al. (2003)	Community residents, aged 19 and older (541)	– Cross-sectional study – To determine health promotion practice	– HPLP II (Spanish version)	Perceived health status, demographics, and acculturation explained 12% of the variance in overall health-promoting lifestyle.
Han et al. (2005)	Adult outpatients in a university hospital, aged 18 and older (436)	– Cross-sectional study – To represent the QOL of patients with CVD	– HPLP – Commitment to a plan of action – Perceived benefit of action	The model explained 63% of the variance in QOL.
Williams et al. (2004)	Low-income African American women (LAAW) (160/134)	– To test a worksite CVD risk factor reduction intervention	– Outcome: risk-reduction behavior	Post-test changes in cholesterol and fat intake risks were more significant in rural than in urban LAAW (*p* < 0.05).
Shin et al. (2005)	Korean adults with chronic disease (400)	– Cross-sectional study – To test 7 constructs from the HPM	– Exercise self-efficacy scale – Exercise social support scale	The path model accounted for 54% of the variance in commitment to a plan for exercise.
Hwang et al. (2015)	Workers in small workplaces (250)	– Cross-sectional study – To represent risk perception and risk reduction behavior of CVD	– HPLP II (52 items) –Job stress	The predictors of health behavior included education level, perceived general health, greater family function, higher social support, decision latitude, and non-shift work.

*BP, blood pressure; CHD, coronary heart disease; CVD, cardiovascular disease; HPM, health promotion model; HPLP II, health promotion Lifestyle profile II; QOL, quality of life.*

Many previous studies have tested the HPM framework among a variety of populations and in diverse settings. As a whole, these studies have provided evidence for the empirical accessibility of the HPM’s concepts and all relationships expressed therein. For example, the Health Promoting Lifestyle Profile instrument was successfully used to measure the HPM’s concepts. Several studies have also implemented the model in the workplace setting (McCullagh et al., 2002; Hwang et al., 2015; Coulombe et al., 2017; Joseph-Shehu et al., 2019; Voskuil et al., 2019). Further, researchers have predicted certain health-promoting behaviors according to its embedded theory, including those related to exercise, nutrition, interpersonal support, stress management, and the use of hearing protection (McCullagh et al., 2002; Joseph-Shehu et al., 2019).

Williams et al. (2004) used the HPM to test a worksite-based CVD-risk factor reduction intervention among low-income African-American workers employed by small companies. Two worker groups (i.e., urban and rural) exhibited higher or similar pre-test CVD-related risks when compared to a national sample of African-Americans. Specifically, an intervention designed to reduce dietary fat intake and cholesterol values was effective among rural workers (i.e., significant changes in cholesterol and fat intake risks; *p* < 0.05). However, this project only incorporated one HPM component and thus did not test its overall usefulness. Although this study only explored two CVD health-related behaviors (physical activity and diet) using HPM variables, it thereby found that preventive services designed for employees at small worksites may reduce CVD risk-factor disparity.

Another study among employees of large companies (e.g., 250 or more employees) found that perceived health status was positively related to health-promoting behaviors aimed at diet and weight control (Coulombe et al., 2017). Here, perceived personal competence, the definition of health, perceived health status, and perceived control of health accounted for 31% of all variance in the examined health-promoting lifestyle patterns (Coulombe et al., 2017). Successful worksite health promotion and disease prevention programs appear rooted in the assessment of personal perceptions among employees. In this context, empirical testing shows that the HPM is an accessible framework for explaining health promotion. Many studies have also shown that the Health Promoting Lifestyle Profile is an appropriate way of assessing a variety of health-promoting behaviors (Hwang et al., 2015; Seo and Ha, 2019).

Pender et al. (2006) reviewed studies that implemented the HPM in various cultures, thus identifying seven significant concepts that explained health behaviors in over half (McCullagh et al., 2002; Carson et al., 2014). These concepts included prior related behavior, perceived health status, perceived benefits and barriers, perceived self-efficacy, social support, and situational influences. Many researchers thus found significant associations between these concepts and health behavior. However, such results only partially support the HPM’s theory. Further testing is thus needed to demonstrate general validity and predictability.

The HPM has also been tested among samples of blue-collar workers and adolescents through the four psychological variables presented in the original model (i.e., importance of health, perceived health locus of control, health status, and self-efficacy) (Weitzel, 1989; Voskuil et al., 2019). It was found that health status and self-efficacy were the most powerful predictors. Indeed, perceived health locus of control and importance of health were deleted in the revised model. Modifying factors (demographics) were also found to predict health-promoting behavior. Although the results were produced over a decade ago, research has shown that the original HPM is potentially useful for explaining health-related behaviors among blue-collar workers. However, the study omitted some variables when operationalizing the model due to research constraints. Nevertheless, it successfully used five instruments (i.e., multidimensional health locus of control, value survey, health status, self-efficacy, and the Health Promoting Lifestyle Profile) to find that most examined variables were highly important for predicting health-promoting behavior (Weitzel, 1989). Like others that did not fully test the HPM, however, these studies only partially support its theory.

The studies discussed here tested variables from the original model or used self-efficacy and perceived health status to explain health-related behaviors. These variables may have been selected because they are easily measured and powerfully predictive. However, the new variables presented in the revised model have not been thoroughly tested. This may be due to the difficulties associated with measuring the concepts of “commitment to a plan of action” and “immediate competing demands and preference” and/or the lack of funding needed to conduct model testing. Clarity and consistency underpin accessibility because these concepts cannot easily be operationalized if they are not clearly defined. Additional theoretical studies are thus needed to implement reliable and valid instruments designed to identify important variables that predict health-related behavior among workers. Future research should therefore aim to determine the predictors of health-related behaviors. The revised HPM also requires full empirical testing.

#### Importance

The HPM has the potential to influence nursing actions such as worksite interventions, health promotion programs, and general education (Voskuil et al., 2019). It may also hold importance for many areas of nursing practice and research in regard to health-promotion behaviors (McCullagh et al., 2002). Considering the emerging ecological perspective on the determinants of health behavior, the environment should also be the subject of more intensive systematic study as a behavioral determinant. In this context, the HPM may have important functions in the development of more effective factors that facilitate health-promoting behaviors. Scholarly efforts should thus be directed at determining the extents of situational influences.

## Discussion

This study comprehensively evaluated and reviewed the HPM. Its underlying theory was thoroughly reviewed to specifically explore the rationale for incorporating the variables of risk perception and risk-reduction behavior into a modified framework for use among blue-collar workers. The HPM provides theoretical guidance for understanding factors associated with health-promoting behavior, including those involved in situational influences and cognition. The model proposes that individuals are more likely to commit to and engage in health-promoting behaviors when they anticipate personally valued benefits and are sufficiently competent to perform a given prospective behavior. The model also posits that situational influences in the external environment can either increase or decrease health-promoting behavior.

The perception of personal risk in relation to disease may be particularly important for risk-reduction behaviors. Specifically, individuals are likely to take recommended health actions if they perceive themselves to be at risk of serious disease. In this context, previous research suggests that the perceived threat of CVD is positively related to the desire to adopt risk-reduction behavior and implement actual behavior changes (Amarasekara et al., 2016; Vetter et al., 2016). As a central construct in many health theories, risk perception is based on an individual’s assessment of their own health situation (i.e., realistic, optimistic, or pessimistic viewpoints). Both optimistic and pessimistic biases have critical implications for illness prevention and disease management. As such, individuals who underestimate their risks are more likely to disregard symptoms and warnings because they believe such indications are more applicable to other individuals. Studies have also clearly shown that blue-collar workers tend to have low CVD-risk perceptions (Fahs et al., 2013; Al-Tamimi et al., 2017; Fox, 2019). Although recent studies have identified CVD risk perceptions among workers in general (Hwang et al., 2012), this is not well described among blue-collar workers. It is therefore important to include CVD-risk perception as a cognitive perceptual factor in the HPM.

We also identified connections among the cognitive, psychological, and situational influence components that affect health behaviors. We then attempted to explore the roles of risk reduction and potential for using the HPM to examine risk-reduction behavior related to CVD among blue-collar workers. However, the HPM seems more applicable for examining health-related behaviors among these workers. First, it has already been empirically tested through a variety of tools and has been directly used to explain health-related behaviors among workers in general (Lagerweij et al., 2018). Further, the HPM has produced stronger and more consistent results when used to test health-related behaviors. Second, it uses multiple variables as determinants of health-related behavior (Carson et al., 2014; Yeom, 2014) while implementing both cognitive and situational components, thus allowing researchers to better integrate underlying environmental associations. Third, the HPM is of sufficient breadth to deal with health-promoting and health-protective behavior.

Health-protection behavior is focused on disease prevention. Health promotion and protection are both related to risk-reduction behavior. Under some conditions (e.g., the absence of disease), risk-reduction behavior can be treated as including health-promotion behavior (e.g., exercise, diet, and smoking cessation). However, risk-reduction behavior seems closer to health protection than promotion when exhibited by individuals with particular diseases, such as CVD and/or diabetes. For example, exercise methods may differ based on whether a given individual is at an asymptomatic or active disease stage. Health improvements should thus be continuously emphasized across the lifespan; in turn, this may improve overall health outcomes (e.g., risk reduction). Because the HPM encompasses both health-promoting and health-protecting behavior, it is the preferred model for explaining health-related CVD behavior among blue-collar workers (Hwang et al., 2015).

This study’s findings showed that the HPM could be used as a foundation for a framework designed to explain risk-reduction behavior among workers. As such, the next section discusses a modified framework that includes individual and environmental characteristics, behavior-specific cognitions and affect, behavioral outcomes, and the concept of risk perception.

### Modified Framework

**FIGURE 3:**
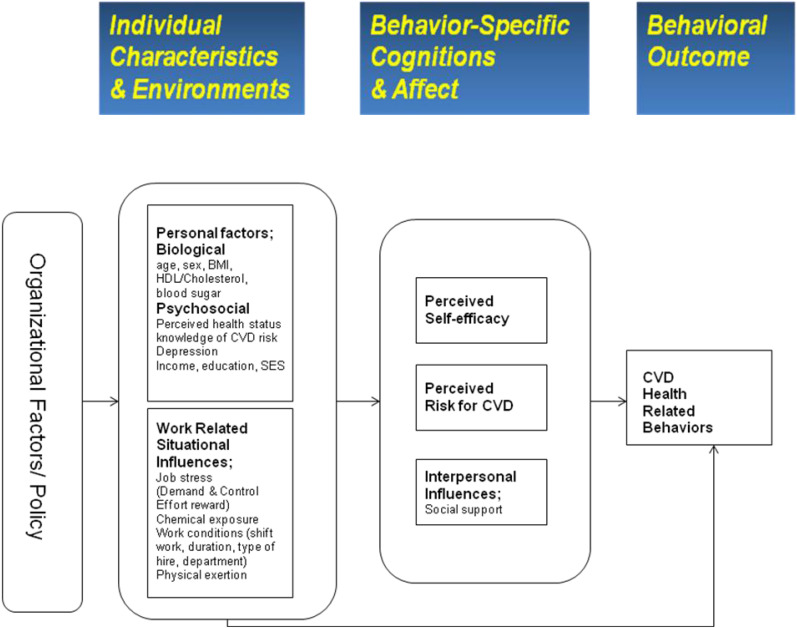
Conceptual framework for CVD health related behavior in blue collar workers. Potential determinants of CVD risk perception and risk-reduction behaviors. Adapted from the Health Promotion Model.

A proposed conceptual framework based on the HPM is shown in Figure 3. This modified model was designed to provide a theoretical framework for CVD-risk perception and CVD health-related behavior research among blue-collar workers.

As Figure 3 shows, the modified framework posits that personal factors and work-related situational influences underlie the construct of individual and environmental characteristics. Here, environmental factors are influenced by public policy and organizational factors (e.g., management style, scheduling, and organizational culture), while work-related situational influences are derived from those found in the HPM; these were restructured under individual and environmental characteristics. Work-related situational influences may affect CVD health-related behavior either directly or indirectly. For example, it is unlikely that individuals with job-related stress will engage in health-related behavior (Chen et al., 2008).

As mentioned, individual and environmental factors may influence health-related behavior either directly or indirectly; this is accomplished through cognition and affect (i.e., perception of self-efficacy, interpersonal influences, and CVD-risk perception). The modified model posits that perceived interpersonal influences, self-efficacy, and CVD-risk perception are the three main cognition and affect factors, while interpersonal influences include perceived social support. CVD health-related behavior may be modified depending on interpersonal influences, such as social support. The model also posits that CVD health-related behavior is influenced by an individual worker’s given CVD-risk perception. It is thus hypothesized that a worker will choose a health-related behavior (e.g., exercise, low-fat diet, weight control, or smoking cessation) if their perceived level of CVD risk is high. Finally, the outcome of a given CVD health-related behavior may provide feedback on behavior-specific cognitions and affect. Due to the added complexity, however, this feedback is not depicted in the modified framework.

#### Concepts

The modified framework comprises the six following key concepts: Individual factors, work-related situational influences, perceived self-efficacy, perceived CVD risk, interpersonal influences, and CVD health-related behavior. The concept of CVD health-related behavior further encompasses physical activity, weight control, and stress management. CVD health-related behavior may be motivated by the desire to protect health by avoiding CVD or increasing one’s level of health regardless of whether CVD is present. Health-related behavior is generally focused on reducing health risks by decreasing the probability of illness through active risk reduction and the detection of health problems during asymptomatic stages.

The concept of personal factors refers to unique biological and psychosocial characteristics that affect subsequent actions and health-related behavior. Biological characteristics include variables such as age, sex, ethnicity, body-mass index values, blood tests (HDL/cholesterol, blood sugar), and known CVD risk factors, while psychosocial characteristics may include variables such as one’s personal knowledge of CVD risk, perceived health status, education, income, and socioeconomic status.

The concept of work-related situational influences refers to workplace environmental factors such as job demand, decision latitude, effort-reward imbalance, coworker support, chemical exposures, physical exertion, and working conditions (e.g., shift work, type of hire, and duration). This may constitute the key to understanding CVD health-related behavior in the workplace. However, the definition of work-related situational influences in this modified model are more closely related to work environments when compared to those originally suggested in the HPM. This is because blue-collar workers may experience job stress and effort-reward imbalances. Here, job stress may negatively influence health-related behavior if, for example, those experiencing such stress are less likely to engage in physical activity other than work (Chen et al., 2008).

The concept of risk perception was derived from perceived threat as expressed in the Health Belief Model (HBM). However, the definition used in this study’s modified model was specifically designed to consider CVD because blue-collar workers are at particularly high risk for that illness, cardiovascular disease. Further, risk perception is a key motivator for CVD health-related behavior (Brewer et al., 2007; Fox, 2019). In this context, CVD risk perception was defined as the level at which blue-collar workers perceived their likelihood of experiencing a CVD event. Risk perception is further influenced by one’s knowledge of CVD risk (Amarasekara et al., 2016; Haynes et al., 2019). The concept of interpersonal influences also includes social support. For example, social support may increase physical exercise among workers. In addition, the concept of self-efficacy is thought to directly influence CVD health-related behavior (Voskuil et al., 2019).

### Assumptions and Propositions

The modified model proposed in this study assumes that individuals actively seek to regulate their own behavior by relying on valued goals. It also entails that personal factors, work-related situational influences, perceived self-efficacy, and interpersonal influences affect CVD health-related behavior. Specifically, CVD risk perception is believed to directly influence CVD health-related behavior. In this model, work-related influences particularly entail the impact of occupational factors in explaining health-related behavior. Thus, the central proposition for testing is that blue-collar workers are more likely to engage in health-related behavior when they perceive CVD risk, believe in their ability to act (self-efficacy), and have assistance and support that enables health-related behavior in the workplace. Perceived CVD risk is also likely to increase when both job stress and the effort-reward imbalance increase, although these factors may also impede appropriate health behavior.

In sum, the key concepts and sub-concepts include biological factors (e.g., age, sex, ethnicity, BMI, HDL/cholesterol, and blood sugar), psychosocial factors (e.g., personal knowledge of CVD risk, perceived health status, education, income, and SES), work-related situational influences factors (e.g., job stress, effort-reward imbalance, chemical exposures, physical factors, and work conditions), perceived self-efficacy, perceived CVD risk, and social support (e.g., social support from coworkers and supervisors). These concepts should be further investigated to determine their specific relationships to risk-reduction behavior among blue-collar workers.

The modified HPM framework can be used to clarify health-related behaviors among blue-collar workers. Also, the revised HPM shows strengths in both accessibility and generality.

This was the first model to consider the impact of both work-related situational influences and risk perceptions on health-related behavior among blue-collar workers. Nevertheless, there are several limitations. For one, both the concepts of work-related influences and risk perceptions may be difficult to operationalize for taking measurements. As such, a prospective study may be required to observe how these concepts impact health-related behavior. Second, other sub-concepts, demand and control, and the effort-reward imbalance must be clarified. The consideration of other factors related to workplace organization and public policy may also have important implications for occupational research. Lastly, the investigation is not based on evidence-based research. The modified framework and suggested concepts should be further investigated to determine their specific relationships to risk-reduction behavior among target populations.

## Conclusion

This study found that risk perception likely influences the health-related behavior adopted by blue-collar workers. This study addresses the modified HPM integration into the current understanding of the problem and how this advances the current views. This study also speculates on the future direction of the research and freely postulates theories that could be tested in the future.

The HPM was theoretically evaluated and reviewed in this context. Because it lacks a critical risk-perception concept, this study’s findings suggest that the original HPM is not suitable for studying risk-reduction behavior among blue-collar workers. The HPM was modified to address deficiencies in regard to the concept of “risk perception.” “Work-related situational influences” were also restructured to underlie the proposed individual and environmental characteristics. Therefore, the modified framework can be used to clarify health-related behaviors among blue-collar workers. Also, the revised HPM shows strengths in both accessibility and generality.

## Data Availability Statement

The datasets generated for this study are available on request to the corresponding author.

## Author Contributions

WH directed this study and collected the data. WH and MK analyzed and interpreted the results, and wrote the manuscript. All authors participated in the design of this study, read and agreed to the final manuscript.

## Conflict of Interest

The authors declare that the research was conducted in the absence of any commercial or financial relationships that could be construed as a potential conflict of interest.
